# The Usefulness of Elastin Staining to Detect Vascular Invasion in Cancer

**DOI:** 10.3390/ijms242015264

**Published:** 2023-10-17

**Authors:** Jeffrey Gonzalez, Hisham F. Bahmad, Stephanie Ocejo, Alvaro Abreu, Meagan Popp, Samantha Gogola, Vielka Fernandez, Monica Recine, Robert Poppiti

**Affiliations:** 1 Herbert Wertheim College of Medicine, Florida International University, Miami, FL 33199, USA; jgonz1074@med.fiu.edu (J.G.); socej001@med.fiu.edu (S.O.); aabre070@med.fiu.edu (A.A.); mpopp002@med.fiu.edu (M.P.); sgogo002@med.fiu.edu (S.G.); 2 Arkadi M. Rywlin M.D. Department of Pathology and Laboratory Medicine, Mount Sinai Medical Center, Miami Beach, FL 33140, USA; vielka.fernandez@msmc.com (V.F.); monica.recine@msmc.com (M.R.); robert.poppiti@msmc.com (R.P.); 3 Department of Pathology, Herbert Wertheim College of Medicine, Florida International University, Miami, FL 33199, USA

**Keywords:** lymphovascular invasion, angioinvasion, venous invasion, TNM classification, staging, immunohistochemistry, elastin stain, College of American Pathologists, World Health Organization

## Abstract

Tumor prognosis hinges on accurate cancer staging, a pivotal process influenced by the identification of lymphovascular invasion (LVI), i.e., blood vessel and lymphatic vessel invasion. Protocols by the College of American Pathologists (CAP) and the World Health Organization (WHO) have been established to assess LVI in various tumor types, including, but not limited to, breast cancer, colorectal cancer (CRC), pancreatic exocrine tumors, and thyroid carcinomas. The CAP refers to blood vessel invasion as “angioinvasion” (vascular invasion) to differentiate it from lymphatic vessel invasion (lymphatic invasion). For clarity, the latter terms will be used throughout this review. The presence of lymphatic and/or vascular invasion has emerged as a pivotal prognostic factor; therefore, its accurate identification is crucial not only for staging but also for providing the patient with an honest understanding of his/her prognosis. Given the prognostic importance of the correct identification of LVI, specific staining techniques are employed to distinguish lymphatic vessel invasion from angioinvasion and to differentiate true LVI from artifact. These encompass hematoxylin and eosin (H&E) staining, elastic staining, Factor VIII staining, Ulex europaeus I agglutinin staining, CD31, CD34, D2-40, ERG, and D2-40 (podoplanin) immunohistochemical (IHC) stains among others. Based on a review of numerous publications regarding the efficacy of various methods for LVI detection, elastin staining demonstrated superior accuracy and prognostic value, allowing for more targeted treatment strategies. The clinical significance of accurately detecting LVI cannot be overstated, as it is strongly linked to higher cancer-related mortality and an increased risk of tumor recurrence. This review aims to examine the existing literature on the use of elastin stains in the detection of vascular invasion among different types of tumors and its prognostic value.

## 1. Introduction

Assessing the prognosis of tumors starts with their cancer staging, a pivotal process aimed at determining the extent of the spread of the tumor itself and its metastasis [1]. The accurate assessment of a tumor’s stage plays a decisive role in prognostication. This is due to the fact that the treatment approach hinges upon whether the tumor’s growth remains localized or has progressed to a disseminated state. The distinction between these scenarios dictates whether the medical team should prioritize curative treatment, such as surgical resection, radiation therapy, and/or chemotherapy interventions, which are more pertinent in the early stages of the disease, or palliative care strategies, which are typically more relevant in the advanced stages [2]. The identification and evaluation of tumors may be initiated by identifying the type of tumor histologically and then determining the stage of the tumor; these characteristics allow of an assessment of their invasiveness into adjacent tissues and the concurrence of metastasis [3]. This categorization is instrumental in shaping treatment strategies and management protocols. Consequently, the staging process is instrumental in gauging the extent of cancer progression within the body [1].

The widely employed framework for staging malignant tumors is the Tumor Node Metastasis (TNM) classification system, which is a universally recognized guideline that facilitates consistent clinical decision-making and prognostic anticipation [4]. This system is based on three principal components: tumor, nodes, and metastasis. The “tumor” element primarily characterizes the dimensions (size) and extent (depth of invasion) of the primary tumor. This classification spans from T0, indicating an absence of detectable tumor, to T4, denoting extensive invasion. The “nodes” component assesses the involvement of adjacent lymph nodes in relation to the tumor and spans the spectrum from no nodal spread to limited local dissemination or more distal spread. In this context, nodal involvement signifies the invasion of tumor cells into lymphatic vessels. Meanwhile, the “metastasis” component evaluates the concurrence of metastatic dissemination to sites remote from the primary tumor other than lymph nodes [4]. Notably, for tumor cells to disseminate from their initial site of development and metastasize to remote sites, vascular invasion is recognized as a prerequisite [5].

Among the aforementioned TNM components, the evaluation of lymphovascular invasion (LVI) constitutes a pivotal factor in the staging process. LVI is considered when malignant cells are identified within lymphatic and/or blood vessels [6,7]. The spread of cancer cells into lymphatic and/or vascular spaces signifies a momentous event and can serve as an indicator of a more aggressive malignancy that has transcended its original location. This process, seen microscopically, is mediated through the recruitment of different chemokines involved, in addition to integrins specific to the infiltrating tumor cells. In accordance with this process, LVI is also mediated by different signaling pathways involved in tumor metastasis. With the increase in different signaling mechanisms, the resulting finding on microscopy is the decrease in endothelial integrity with increased permeability to tumor cells. Overall, collapsed vessels, endothelial junction damage, increased permeability, and staining of different tumor markers within these vessels are observed when analyzed microscopically [8].

Within the realm of LVI, a multitude of categories exists, encompassing lymphatic, arterial, and venous vessel invasions. The process of LVI transpires as tumor cells obtain access to lymphatic vessels and/or blood vessels, typically through breaches in the epithelial basement membrane, subsequently invading the surrounding tissue [9]. Of note, the invasion into blood vessels assumes greater significance due to its correlation with an adverse prognosis, potentially facilitating the metastatic dissemination of the tumor to distant anatomical sites, whereas invasion into lymphatic vessels may potentially prompt lymph node involvement [9]. Extensive research underscores the correlation between the infiltration of blood vessels and/or lymphatics by tumors and unfavorable patient outcomes, particularly evident in cases of colorectal cancer (CRC) [10,11,12,13,14]. Overall, a prevailing consensus prevails: the presence of LVI is correlated with worse outcomes across various malignancies [7,15,16,17,18].

**Figure 1:**
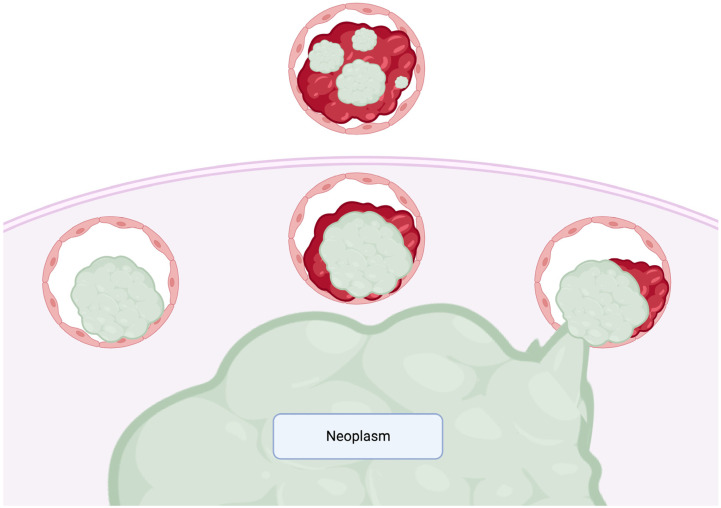
Schematic diagram for the interpretation of the presence of vascular invasion. The diagram illustrates a neoplasm (green) surrounded by a fibrous pseudocapsule (tan). For vascular invasion to be considered true invasion, the neoplastic cells should either penetrate through the vessel wall or should instigate a reaction to the vascular deposit via thrombus formation. Left to right: tumor deposit juxtaposed to the vessel wall (technically, although some pathologists may not count this as significant vascular invasion; this scenario is considered a “judgmental call”); tumor deposit juxtaposed to the vessel wall and associated with a thrombus; tumor cells directly penetrating the vessel wall and demonstrating thrombus formation. Above: tumor deposits within an organized thrombus, adherent to the vessel wall, external to the fibrous pseudocapsule of the tumor.

To establish a more standardized approach for reporting and comprehending tumor characteristics, the College of American Pathologists (CAP) and the World Health Organization (WHO) have integrated means to incorporate the evaluation of LVI into tumor staging. This integration is exemplified through the CAP cancer protocols and the “WHO Classification of Tumors”. The CAP protocols encompass an evaluation of various tumor types, such as invasive breast carcinoma, CRC tumors, and pancreatic exocrine tumors, specifically assessing LVI [19,20] (Figure 1). As delineated by the CAP, vascular invasion entails the presence of blood vessels located externally to the tumor, within its encapsulation, or outside of the tumor’s fibrous pseudocapsule [10,21]. The assessment of vascular invasion can present challenges, particularly when dealing with diminutive, capillary-sized vessels. In such instances, the application of markers selective for the endothelium of vascular and lymphatic spaces, such as CD31 and podoplanin, has proven instrumental [22,23,24,25]. Amid ongoing debates surrounding the definition of LVI, the CAP has adopted more precise and rigorous criteria, characterizing the process as the infiltration of cancerous cells through the endothelial membrane and into the vessel lumen. This criterion, as endorsed by the CAP, has demonstrated significant predictive utility in identifying more aggressive malignancies, as exemplified in thyroid tumors [26].

This review aims to examine the existing literature on the use of elastin stains in the detection of vascular (particularly venous) invasion among different types of tumors and their prognostic value. PubMed served as our main search database. All articles were selected from peer-reviewed journals. Articles included in this review were selected based on their relevance to the topic discussed: various approaches employed in detecting vascular invasion across different tumors and elastin stain usage to detect venous invasion in CRC and other malignancies.

## 2. Various Approaches Employed in Detecting Vascular Invasion

As outlined by the guidelines set forth by the CAP, microscopic vascular invasion is defined by the presence of tumor cells within a vascular space lined by endothelium [27]. For hepatocellular carcinoma (HCC), such identification is feasible exclusively through microscopic examination within the tumor’s pseudocapsule, noncapsular fibrous septa, or the surrounding tissue [27]. The validation of vascular invasion necessitates the observation of tumor attachment to the vessel wall or the discernment of smooth muscles or elastic lamina [27]. Moreover, the identification of an intact vessel juxtaposed with tumor cells and concomitant thrombus formation serves as a valuable criterion in distinguishing true vascular invasion from potential artifacts. Instances involving non-thrombosed tumor cells within a compromised vascular lumen are most likely attributed to tissue displacement incurred during the slide preparation process [26]. In this context, distinctive features useful for distinguishing between artifacts and true vascular invasion within hematoxylin and eosin (H&E)-stained slides encompass the “orphan arteriole” and “protruding tongue” signs. The concept of an “orphan arteriole” generally refers to a tumor nodule adjacent to a muscularized artery in the absence of an accompanying vein. On the other hand, a “protruding tongue” sign generally refers to an even ended and rounded protrusion into the nearby pericolic fat located in close proximity to a blood vessel, chiefly an artery [28].

**Figure 2:**
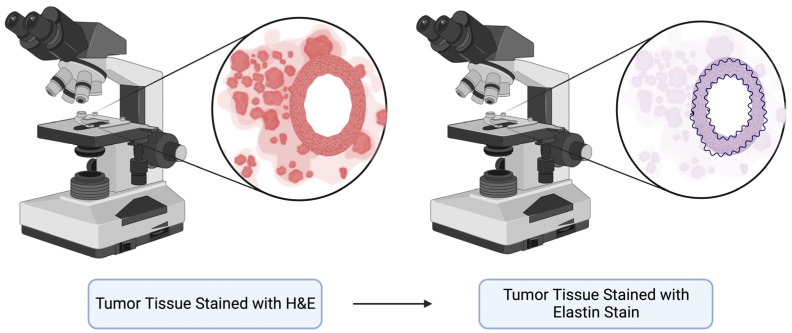
Schematic diagram depicting the benefit of using elastic staining to visualize vascular invasion in tumor tissue samples.

The presence of intravascular tumors that may be adjacent to a vessel wall can sometimes lead to the determination of certain paths of treatment due to the risk of vessel invasion and the possibility that a resection of the tumor in patients with the invasion of an adjacent vessel may be curative [29]. While the H&E stain does allow for the visualization of endothelialized spaces, it is limited in the ability to differentiate angioinvasion from lymphatic invasion [5]. Moreover, it is crucial to differentiate intraluminal invasion from vascular wall invasion, as this distinction can impact the staging of certain tumors. Vascular wall invasion is characterized by tumor penetration through the vascular wall or the initiation of a reaction in the vessel wall, whereas intraluminal invasion may involve free-floating tumor fragments or endothelialized tumor fragments [25]. As an example, in the context of HCC, intraluminal invasion does not have the same impact on tumor staging as vascular wall invasion [25]. While the CAP underscores the use of elastic staining or immunohistochemistry (IHC) targeting smooth muscle when interpreting specimens (Figure 2), it is noteworthy that several other staining techniques are employed for the detection of tumor-associated vascular invasion.

**Figure 3:**
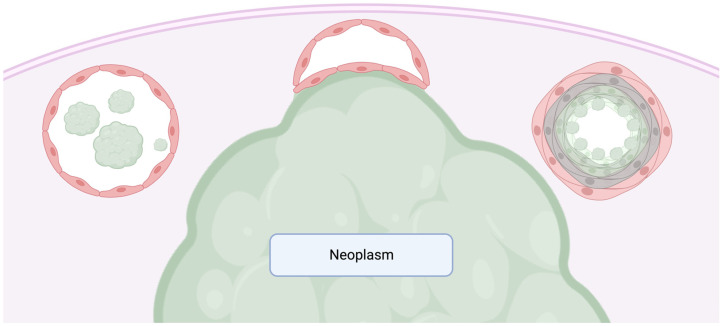
Schematic diagram for the interpretation of pitfalls in assessing vascular invasion. The diagram illustrates a neoplasm (green) surrounded by a fibrous pseudocapsule (tan). Three scenarios are presented from left to right where tumor in/abutting the vessel should not be counted as true vascular invasion: free-floating tumor fragments within the vessel lumen (likely a result of artifactual displacement); tumor bulging and compressing/abutting the vessel wall externally; endothelialized tumor cells floating withing the vessel (may be a result of the tangential sectioning of a tumor bulging into a vessel) which prompt taking deeper sections to exclude or include definitive vascular invasion.

Many cases of LVI may pose challenges in their identification; however, the distinction between true vessel invasion and artifacts remains vital for informed care management. Certain scenarios warrant cautious discernment to exclude them from being categorized as LVI, including free-floating tumor fragments, a tumor bulging into a vessel causing its distortion, and the presence of endothelialized tumors within intracapsular vessels [26] (Figure 3). For example, cases which may introduce challenges in the identification of true LVI include CRC. In such cases, there is no consensual and reliable means by which accurate discernment between true angioinvasion or lymphatic vessel invasion and artifact can be made through the use of the H&E stain alone or through the use of endothelial markers such as CD31 and CD34 [30]. It is noteworthy that, aside from artifacts arising from slide preparation or morphological alterations, desmoplastic reactions and tumor-invaded endothelial destruction can compromise vessel walls, rendering the recognition of the original vascular anatomy notably difficult.

Although the true prognostic significance of detecting blood and/or lymphatic vessel invasion in some tumors continues to be debated, a consensus is emerging that LVI in general holds an independent prognostic value, indicative of heightened potential for distant metastasis and poorer survival rates [31,32,33]. To illustrate, the CAP elevates the staging of HCC from pT1 to pT2 in the presence of vascular invasion, underscoring the indispensability of identifying vascular invasion to formulate optimal treatment strategies [26]. Llovet et al. demonstrated that the identification of vascular invasion in HCC could even prompt the initiation of palliative interventions aimed at enhancing survival, such as chemoembolization [34]. Furthermore, the CAP emphasizes the significant roles of angioinvasion (vascular invasion) in follicular thyroid carcinoma and lymphatic invasion in papillary thyroid carcinoma.

Notably, recent studies have also highlighted the prognostic significance of the number of LVI invasion foci. For instance, a study conducted by Stojadinovic et al. concluded that follicular thyroid carcinoma with four or more foci of vascular invasion had a significantly higher recurrence rate, even when some of the foci were microscopic in nature [35]. Yet, despite the presence or absence of vascular invasion, the standard therapeutic approach to follicular thyroid carcinoma typically involves total thyroidectomy and post-operative radioactive iodine (RAI) therapy. Analogously, in the context of CRC, the presence of vascular invasion may prompt the inclusion of adjuvant therapy [28]. Given that the treatment plans are apt to be restructured based on vascular invasion identification, surmounting the challenges associated with accurate vascular invasion diagnosis assumes pivotal significance in augmenting patient survival and overall disease-free life expectancy.

An array of staining techniques of varying efficacy can be employed to assess LVI, encompassing the H&E stain, elastic staining, Factor VIII staining, Ulex europaeus I agglutinin staining, CD31, CD34, D2-40, ERG, and D2-40 (podoplanin) IHC stains among others. While H&E staining facilitates the recognition of structural anomalies, alternative staining methods have gained importance for enhancing and supporting LVI detection. Notably, the Elastica Van Gieson stain serves to visualize elastic fibers within connective tissue, proving valuable not only in identifying vascular invasion but also in discerning vasculitis or vessel reduplication. Of significance, elastic fibers are present in the adventitia of veins (but not lymphatics) and Elastica stains can be used to highlight the presence of veins and their adjacent arteries, serving as a discriminative feature between lymphatic and vascular invasion and thereby simplifying the differentiation between vascular and lymphatic invasion [36].

### 2.1. Vascular Invasion

#### 2.1.1. Elastin Stain

In a study by Howlett et al., the utilization of Movat’s elastin stain significantly enhanced the accurate identification of venous invasion in CRC, surpassing the conventional H&E stain [13]. Remarkably, this approach led to the reclassification of previously negative venous invasion cases on H&E slides, with a notable 44% of these cases being reclassified as positive when viewed with the Movat stain [13]. This heightened accuracy in identifying venous invasion contributed to refined prognostication, enabling more precise predictions of metastatic potential [13].

A recent study by our group also explored the effectiveness of the routine elastin staining of all tumor-containing blocks on the detection of venous invasion in CRC specimens. We compared 93 specimens not stained with elastin stain to 61 specimens stained with elastin stain. We particularly demonstrated that in instances where vessel distortion or obliteration was present, elastin staining exhibited superior performance. Our findings revealed a substantial enhancement in venous invasion detection rates, with Van Gieson’s elastin staining associated with a notable 4.5-fold increase in venous invasion identification upon implementing routine staining on all tumor-containing blocks compared to H&E alone [11].

Furthermore, in response to a venous invasion detection rate of merely 14% (11% excluding academic hospitals) reported in 2010, a cohort of pathologists in Ontario sought to address the seemingly low venous detection rates by advocating for the increased usage of elastin staining [12]. Remarkably, this initiative yielded noteworthy outcomes, with 72% of pathologists who escalated their routine application of elastin staining acknowledging a perceptible enhancement in their ability to detect venous invasion [12].

In a study by Kirsch et al., a comprehensive analysis encompassed 80 CRC specimens, venous invasion was assessed in 40 specimens using H&E alone, and 40 cases were assessed using H&E and Movat’s elastin stain for venous invasion identification. The findings were striking, revealing that the detection of venous invasion was more than twofold higher with the application of Movat staining in comparison to relying solely on H&E staining [37]. Collectively, a robust body of evidence underscores how elastic staining has substantially augmented the accuracy of identifying true venous invasion [13,32].

While various options exist for identifying vascular invasion, some have demonstrated greater efficacy in discerning true invasion from artifacts. For instance, the identification of vascular invasion through H&E staining is directly correlated with the number of slides examined, which consequently increases the time and resources invested in the review process [10,28]. In this regard, Messenger et al. highlighted that the adoption of elastin stains for venous invasion identification in CRC led to decreased diagnostic time and a more streamlined utilization of blocks, ultimately contributing to a more efficient diagnostic process [28]. Similarly, we have shown in a recent study by our group that implementing routine elastin staining on all tumor-containing blocks, rather than on selected tumor-containing blocks, increased the venous invasion detection rate in CRC [11].

#### 2.1.2. Factor VIII Stain

Factor VIII staining, although used by some for vascular invasion detection, has demonstrated limited utility [38]. In a study of ten cases involving follicular carcinoma of the thyroid, staining with factor VIII-related antigen exhibited weak staining of vessels within the tumor and an absence of staining in vessels occluded by the tumor. However, vessels only partially occluded by the tumor exhibited staining [38]. Based on the absence of staining within the tumor, the authors concluded that factor VIII-related antigen might not prove advantageous in vascular invasion identification [38].

#### 2.1.3. CD31 and CD34 Immunohistochemical Stains

CD31 and CD34 serve as markers targeting vascular endothelial cells. CD31 exhibits a distinct advantage over CD34, as it generates less background staining by predominantly highlighting endothelial-lined tumor emboli, thereby facilitating the more straightforward identification of tumor cells invading the vasculature [39,40]. In a study conducted by Lin et al., an examination of 32 follicular thyroid carcinoma specimens included the application of H&E, D2-40, CD31, and CD34 staining [39]. This investigation revealed that out of 32 cases, almost one third (13 cases) were detected to have vascular invasion using H&E alone, while D2-40 identified none, and each of CD31 and CD34 identified 14 cases with vascular invasion [39]. Although the percentage of tumors identified to have vascular invasion with CD34 and CD31 was comparable to that of H&E alone, Lin et al. unequivocally advocate for the preference of CD31 over CD34 and D2-40 in the identification of vascular invasion for follicular thyroid carcinoma due to the potential of CD34 to stain non-endothelial cells [39].

Kurtz et al. undertook a study involving 40 cases of oral cavity squamous cell carcinoma (SCC) wherein the vascular invasion status had previously been reviewed. All slides underwent a secondary review (by H&E) and were subsequently stained with CD31. The original review unveiled vascular invasion in 30% of the slides, with the second review identifying vascular invasion in 35%, while CD31 staining led to the identification of vascular invasion in 42% of the cases. The discovery of vascular invasion in these patients exhibited a high correlation with mortality at the 5-year follow-up [41]. Comparing the staining methods of Elastica Van Gieson, CD31, and CD34 in CRC specimens, Kingston et al. examined 50 archival tumor sections. The results indicated that the identification of vascular invasion was significantly augmented using Elastica Van Gieson (24 cases), CD31 (18 cases), and CD34 (21 cases) compared to relying solely on H&E staining (5 cases) [42].

#### 2.1.4. Ulex Europaeus I Agglutinin (UEAI) Staining

Ulex europaeus I agglutinin (UEAI) staining constitutes another strategy employed to detect vascular invasion, particularly in the context of urothelial carcinoma (formerly transitional cell carcinoma; TCC) of the bladder [43]. Larsen et al. conducted a restaining endeavor using UEAI on 36 H&E-stained TCC specimens with remarkable outcomes. This approach facilitated the differentiation between “pseudoinvasion”, potentially attributable to retraction artifact, and true vascular invasion [43]. Notably, UEAI staining in endometrial carcinoma emerged as a direct correlate of patient survival, with high UEAI staining coinciding with high-grade carcinomas exhibiting vascular invasion tendencies [44]. Furthermore, caution has been voiced regarding potential cross-reactivity of factor VIII-related antigen with structures such as Weibel–Palade bodies and mitochondria, which may impact the stain’s efficacy [45].

### 2.2. Lymphovascular Invasion

#### 2.2.1. D2-40 (Podoplanin) Immunohistochemical Stain

From a physiological standpoint, D2-40 (podoplanin) plays a significant role in increasing endothelial cell adhesion and serves as a marker for lymphatics and lymphatic differentiation within vascular tumors [46]. D2-40 has exhibited clarity in demarcating tumor emboli within the lymphatics or primary tumors of various sites, including the breast, colon, prostate, cervix, endometrium, and skin, and in distinguishing vascular and lymphatic invasion since it is not expressed by vascular endothelial cells [47]. A comparative study involving 50 breast cancer cases indicated that D2-40 staining enabled the identification of lymphatic invasion in 44% of lymph node-negative cases and an impressive 88% of lymph node-positive cases. Notably, the study revealed that 18% of H&E specimens yielded false-negative results, while 4% yielded false-positive results [47].

Schoppmann et al. extended the analysis of podoplanin staining to breast cancer cases, reporting the successful identification of lymphatic microvessel density and LVI within breast cancer specimens [48]. The identification of LVI in breast cancer emerged as a pivotal prognostic factor, notably impacting the prediction of lymph node metastasis and paving the way for more tailored treatment regimens [48].

Studies by El-Gohary and colleagues demonstrated increased mean periductal D2-40 lymphatic microvessel density and CD31 microvessel density in cases of high-grade intraductal carcinoma suspicious for invasion, concluding that that lymphatic and blood vascular densities evaluated by D2-40 and CD31, respectively, were independent prognostic indicators for patients with intraductal carcinoma of the breast [49,50].

Additionally, Mohammed et al. conducted a comparative analysis of CD31, CD34, and D2-40 (podoplanin) in the identification of vascular invasion within 177 breast cancer specimens. Their findings indicated what while CD31 and CD34 exhibited strong positive staining in vascular vessels, they had variable detection of lymph vessels [51]. In contrast, D2-40 demonstrated a lack of reactivity in blood vessels but displayed strong affinity for lymphatic vessels, thereby facilitating the improved visualization of lymphatic vascular invasion [51]. The use of all three stains led to a notable increase in the detection of LVI compared to the standard H&E staining, and it enhanced the level of agreement among pathologists in distinguishing between lymphatic and vascular invasion [51]. In a subsequent review, the authors found that while CD34 non-specifically binds to stromal components, potentially contributing to an increase in false positives, they strongly advocate for the adoption of specialized staining techniques to identify vascular invasion [52].

#### 2.2.2. ERG Immunohistochemical Staining

Finally, the staining for ERG, a remarkably specific endothelial marker, holds promise due to its high specificity for LVI [53,54]. A study by Kim et al. analyzed fifteen cases of surgically resected CRC tumors with hepatic metastasis, representative of LVI, and subjected them to ERG, CD31, and D2-40 staining. The outcomes demonstrated that although the LVI positivity rates were highest with H&E alone (43%), ERG staining proved superior to CD31 and D2-40 in detecting LVI [54]. ERG immunostaining showed distinct nuclear immunoreactivity of endothelial cells in the artery, vein, and lymphatic vessels without cross immunoreactivity compared to CD31 stainability in inflammatory cells and D2-40 stainability in fibroblasts. Intriguingly, pathologists exhibited enhanced consensus agreement with ERG staining due to its capacity for improved visual contrast in detecting LVI [54].

## 3. Elastin Stain Usage to Detect Venous Invasion in Colorectal Carcinoma and Other Malignancies

### 3.1. The Use of Elastin Stain in Colorectal Carcinoma

The detection of venous invasion holds paramount significance in CRC prognosis. Numerous studies have assessed the utility of elastin stains in enhancing the detection of venous invasion, as depicted in Table 1. A study conducted in Toronto, Canada, revealed a marked increase in venous invasion detection from 20% to 45% following the implementation of elastin staining [14]. Impressively, the miss rate for venous invasion decreased from 48% to 22% with elastin staining [14]. Furthermore, a statistically significant association was found between venous invasion detection via elastin staining and an elevated risk of hematogenous spread, decreased relapse-free survival, and reduced cause-specific survival [14]. Similarly, a study by our group yielded a rise in the venous invasion detection rate in the elastin-stained cohort (50.8%) compared to H&E staining alone (18.6%) [11]. Supporting these findings, a survey encompassing 361 Canadian pathologists documented a twofold increase in venous invasion detection upon the incorporation of elastin stains [12].

Howlett et al. examined venous invasion detection and its prognostic implications. Although the detection of venous invasion was significantly increased upon elastin stain utilization, the latter had no effect on prognosis [13]. Conversely, Roxburgh et al. reported a 96% 3-year cancer-specific survival rate among those negative for venous invasion using an elastin stain, whereas only 84% survived among those negative for venous invasion with H&E alone [55]. Strikingly, only venous invasion identified through an elastin stain proved predictive of worse survival outcomes [55].

A pivotal aspect of venous invasion detection lies in the morphology of the tumor itself. Hwang et al. demonstrated that venous invasion detection increased significantly from 15.1% in the initial pathology report to 40.9% when pathologists focused on carefully looking for venous invasion on H&E slides alone and further to 48.4% with elastin staining [56]. Although specifically looking for venous invasion increased the detection rate on H&E, using an elastin stain further augmented this rate.

Conclusively, a comprehensive literary review by Messenger et al. disclosed that most studies report a threefold increase in the venous invasion detection rate, even among non-gastrointestinal (GI) pathologists, upon the application of elastin staining [28]. This finding’s significance resonates with the Kirsch et al. study, which assessed the venous invasion detection rates between GI and non-GI-pathologists using H&E-stained samples versus Movat-stained samples. The study concluded an increased detection rate of extramural venous invasion and enhanced interobserver agreement when Movat staining was employed, irrespective of the pathologist’s experience [57].

**Table 1:** Systematic evaluation of elastin staining for detection of venous invasion in various types of tumors.

Author and Ref.	Designs and Participants	Main Findings	Elastin Stains on Venous Invasion Detection and Prognostic Value
Bahmad et al., 2023 [11]	Before-and-after study of CRC Case studied: Patients who underwent resection in the year before the implementation of elastin stain (n = 93) and after the implementation of elastin stain (n = 61)	Venous invasion (H&E alone): 18.6% Venous invasion (elastin stain): 50.8%	Venous invasion detection rate, using an elastin stain, more than doubled compared to H&E alone This study did not correlate its findings to the patients’ clinical outcomes; thus, the prognostic value could not be determined
Sari et al., 2022 [14]	Before-and-after study of CRC Cases studied: Patients who underwent resection in the year before the implementation of elastin stain (n = 144) and after the implementation of elastin stain (n = 128)	Significant increase in the venous invasion detection rate (45% in the post implementation vs. 20% in the pre implementation cohort) Venous invasion (H&E alone): 20% Venous invasion (elastin stain): 45%	Venous invasion detection rate using an elastin stain more than doubled compared to H&E alone by GI and non-GI pathologists
Gopinath et al., 2020 [58]	Retrospective cohort study 105 non-negative primary head and neck SCC cases	Venous invasion (H&E alone): 7% Venous invasion (elastin stain): 35%	Venous invasion detection rate using an elastin stain more than doubled compared to H&E alone In node-negative primary head and neck SCC, venous invasion detection was not a significant prognostic factor for the RFS time or overall survival
Liu et al., 2020 [59]	Retrospective cohort study 104 cases of small intestinal NETs	Extramural venous invasion (H&E alone): 43.3% Extramural venous invasion (VVG): 55.8%	There was a 1.3-fold increase in venous invasion detection after elastin stain implementation Significant association between extramural venous invasion and metachronous liver metastasis
Takada et al., 2020 [60]	Retrospective cohort study of 100 specimens of submucosal gastric carcinoma Three pathologists (expert, intermediate, and trainee) independently evaluated lymphatic invasion and venous invasion status using H&E-stained slides and reevaluated their decisions by reviewing the corresponding D2-40-stained and elastin-stained slides	Venous invasion (H&E alone): 20% for expert pathologists, 20% for intermediate pathologists, and 4% for trainees Venous invasion (elastin stain): 31% for expert pathologists, 33% for intermediate pathologists, and 23% for trainee pathologists	Elastin stain significantly increased venous invasion detection rates among pathologists regardless of experience level
Dawson et al., 2014 [12]	Population-based survey of 361 pathologists	An audit for the year prior to and the year following the implementation of routine elastin staining revealed a twofold increase in venous invasion detection rates (20% vs. 42%) Venous detection rate in the year prior to elastin stain implementation (H&E alone): 20% Venous detection rate in the year after elastin stain implementation: 42%	Twofold increase in the venous invasion detection rate after implementing elastin stain Elastin stains provided a more sensitive methodology for determining venous invasion compared to H&E alone
Kirsch et al., 2013 [57]	Retrospective cohort study of 40 H&E-stained slides and 40 corresponding Movat-stained slides derived from 37 cases of CRC	Among the 12 pathologists, the mean venous invasion detection rate on H&E in this study set was 19.6% The average venous invasion detection rate by GI pathologists was 30% compared with 9.2% among non-GI pathologists Venous invasion (Movat stain): Mean venous invasion detection rate among all 12 pathologists was 46.4%; 58.3% for GI pathologists and 34.6% for non-GI pathologists	The use of an elastin stain showed a significant increase in the overall venous invasion detection rates as well as increased agreement among all pathologists regardless of the level of training.
Messenger et al., 2012 [28]	Literature review highlighting the potential impact of recent developments on future practices with regard to venous invasion assessment	In most studies, a 3-fold increase in the venous invasion detection rate was noted when elastin stain was used, even among non-GI pathologists	Study demonstrated a consensus across the literature on the significant increase in the venous invasion detection rate when elastin stains were used compared to the standard H&E stain Based on the literature, venous invasion, especially extramural venous invasion, serves as an independent prognostic factor and predictor of visceral metastasis
Roxburgh et al., 2010 [55]	Prospective cohort study of 419 CRC patients Patients were grouped as cohort 1 (1997–2001) prior to implementing elastin staining and cohort 2 (2003–2006) following the introduction of elastin staining	Venous invasion (H&E alone): 18% Venous invasion (Elastica stain): 58%	Results showed that the absence of venous invasion detection when using an elastin stain had a higher predictive value for 3-year cancer specific survival than H&E alone Only venous invasion detection through elastin staining was associated with decreased survival
Howlett et al., 2009 [13]	Retrospective cohort study of 92 CRC cases assessed for venous invasion on H&E-stained slides and on Movat pentachrome	Initial review of the H&E-stained slides: 50 cases as negative, 25 as equivocal, and 17 cases as positive for venous invasion Follow-up review using Movat stain: 22/50 (44%) of the “negative” group cases, 19/25 (76%) of the “equivocal” group cases, and 16/17 (94%) of the “positive” group cases stained positive for venous invasion	The use of elastin stain showed a significant increase in the venous invasion detection rate among H&E-stained slides deemed negative or equivocal for venous invasion Elastin staining showed the highest overall venous invasion rate; however, venous invasion detected by elastin stain had the same prognostic value as that detected with H&E with respect to metastasis

Abbreviations: CRC: colorectal carcinoma; CSS: cause-specific survival; FL: Florida; GI: gastrointestinal; H&E: hematoxylin and eosin; SCC: squamous cell carcinoma; NET: neuroendocrine tumor; ON: Ontario; RFS: relapse-free survival; USA: United States of America; VVG: Verhoeff–Van Gieson.

### 3.2. Use of Elastin Stains in Malignancies Other Than Colorectal Carcinoma

The prognostic value of venous invasion in other malignancies is less extensively explored in the literature. Gopinath et al. examined the prognostic implications of venous invasion detection in node-negative head and neck SCC. Similar to CRC studies, venous invasion detection significantly improved with elastin stain utilization. While univariate analysis revealed a statistically significant decrease in recurrence-free survival for patients with venous invasion, this significance did not persist in multivariate analysis [58]. A separate study examined extramural venous invasion in neuroendocrine tumors (NETs) of the small intestine, highlighting a 1.3-fold increase in the detection rates post Verhoeff–Van Gieson (VVG) elastin staining. Notably, extramural venous invasion was found in 100% of cases with liver metastasis compared to 54.5% in non-liver metastasis [59]. Lastly, a study by Takada et al. assessed the impact of D2-40 and elastin staining on interobserver agreement when examining LVI in gastric cancer. The findings concluded that the ancillary use of an elastin stain significantly improved interobserver agreement in venous invasion detection, irrespective of experience [60].

Additionally, elastin stains can provide evidence of structural changes that may not involve venous invasion. An example is the use of elastin stain in pulmonary adenocarcinoma to demonstrate vascular and pleural invasion [61]. Using an elastin stain can also help differentiate between arteries and veins when the histologic sample does not portray apparent determining factors and, thus, invasion by the tumor cells [61]. An elastin stain to assess the pleural tissue is used for staging in pulmonary adenocarcinoma by examining the presence of cancer cells beyond the outer elastic layer or beyond this outer layer and onto the visceral pleura [61]. An important finding in this study was the importance of the elastin tissue structure seen upon staining. The study found that the tissue architecture may already be altered when pulmonary adenocarcinoma arises in the presence of a preexisting condition such as emphysema. If the elastin pattern in the malignancy resembles the preexisting condition, the structural alterations are most likely due to the condition and not the tumor [61]. Elastin stains can also help differentiate hepatic tumors. Determining between segmental atrophy of the liver and sclerosing cavernous hemangiomas can be difficult given that the latter can often mimic the nodular elastosis stage of segmental atrophy [62]. With the use of Verhoeff’s elastin stain, both segmental atrophy and sclerosing cavernous hemangiomas showed some levels of elastin, but only segmental atrophy showed elastin to be densely and diffusely positive in the majority of the samples as opposed to only one of the sclerosing cavernous hemangiomas [62]. These findings suggest that elastin is a useful stain when segmental atrophy is suspected.

A study by Liu et al. assessed the use of elastin stain plus the AE1/AE3 IHC stain to assess the prognostic significance of vascular invasion in pT1b esophageal SCC [63]. The study found that vascular invasion detection using this stain was associated with distant metastasis and adverse prognostic factors for overall survival and recurrence-free survival [63]. The adjunct use of elastin stain with other techniques has also been helpful in the detection of blood vessel invasion in breast cancer. Detecting blood vessel invasion in breast cancer is of limited diagnostic usefulness due to the components of intraductal carcinomas mimicking blood vessel invasion. A study by Fujisawa et al. used a combination stain of Victoria blue for elastin and IHC for collagen type IV [64]. This allowed the differentiation between the tissues of vascular origin, which would show reigned or lost collagen fibers, and the tissues of intraductal carcinoma, which would show a normal arrangement of the collagen in the basement membrane [64]. The results demonstrated a higher degree of blood vessel invasion detection using the combined stain (49%) compared to Van Gieson’s elastin stain alone [63]. Lastly, detecting blood vessel invasion through this technique showed a significant association with worse prognosis. This association was found to be stronger than that seen with lymphatic vessel invasion [64].

## 4. Clinical Value of Detecting Lymphovascular Invasion

In numerous instances, the presence of LVI has emerged as a pivotal factor in delineating the prognosis of patients with various malignancies. LVI serves as a primary conduit for tumors to disseminate from their original site to distant lymph nodes and organs. The detection of LVI can offer insights into survival rates, relapse possibilities, recurrence percentages, and the propensity for subsequent complications associated with the pathology of the tumor itself. An illustrative example lies in cases of gallbladder carcinoma where, after removal of the gallbladder, LVI has been linked to complications such as portal vein hypertension and ruptures of esophageal and rectal varices [65]. Nevertheless, the exact mechanism behind this link is poorly understood. Collectively, numerous studies underscored the pivotal role of LVI in prognosticating survival and recurrence rates across various cancers. Through the early detection of LVI on microscopy, the patient is afforded the possibility of being provided with a more accurate diagnosis, timeline, and treatment plan. Through further research done on LVI, this may also allow for treatment targeting the mediators involved in LVI in the future [9].

LVI exhibits associations with tumor size, mitotic index, TNM stage, and tumor depth [66]. For instance, a prognostic study involving 361 participants who underwent gastrectomy for nonmetastatic gastric cancer revealed that 13.9% of the cohort exhibited vascular invasion. This study established a significant correlation between vascular invasion and tumor size, infiltration depth, and TNM stage, as ascertained through Cox proportional hazard models. Notably, subgroup analyses within the same study unveiled vascular invasion as a predictor of cancer-specific survival, specifically for patients in stage 1 [66]. In patients with gastric cancer, 20–30% experienced distant and local recurrences during follow-up periods. Consequently, the early identification of prognostic factors assumes paramount importance in devising treatment strategies and enabling patients to make informed decisions aligned with their preferences. Furthermore, vascular invasion was linked to a notably diminished cancer-specific survival and disease-free survival compared to patients without vascular invasion. Even after adjusting for tumor size and TNM stage, vascular invasion amplified the risk of cancer-related mortality by 1.7-fold in contrast to patients without vascular invasion [66]. These findings underscore the critical implications of vascular invasion in predicting patient outcomes, ultimately guiding therapeutic interventions and informing patient-centered decisions.

In a retrospective cohort study focusing on pancreatic NETs, a range of prognostic factors emerged alongside venous invasion. Cox proportional hazard analysis, tumor size, Ki-67 proliferation index, mitotic count, and venous invasion were all identified as significant predictors of poor prognosis. Although the sample size was modest, with 9 out of 32 patients exhibiting venous invasion after tumor resection, 5 of these 9 experienced postoperative recurrences. Notably, these 9 patients faced significantly worse prognoses compared to those without venous invasion in terms of tumor recurrence [67]. Interestingly, the study revealed that a tumor size exceeding 20 mm significantly elevated the risk of venous invasion. Moreover, a high mitotic count and Ki-67 proliferation index were also significantly associated with an increased risk of venous invasion [67].

LVI has also emerged as a noteworthy prognostic factor in the context of endometrial cancer. It exhibited associations with higher rates of G2 tumors, myometrial infiltration, and larger tumor dimensions [68]. The influence of LVI as a poor prognostic factor was particularly evident in early-stage endometrial cancer, being predictive of relapse. Patients lacking LVI demonstrated a robust 5-year disease-free survival rate of 93.6% in contrast to 56.5% for those with LVI. The study meticulously selected participants without lymph node involvement or other health risks to ascertain the true independence of LVI as a prognostic determinant. The study further unveiled that substantial LVI, characterized by diffuse or multifocal invasion, independently conferred increased risks of distant recurrences and emerged as the most potent predictor for poor prognosis in terms of disease-free survival and overall survival [68]. This finding underscores LVI’s significance as an independent predictor, emphasizing the importance of LVI detection for effective preparation against potential recurrence and for providing patients with insight into their prognosis.

The detrimental impact of venous invasion on prognosis has been accompanied by the emergence of comorbidities, particularly in cases of HCC. A clinical study delving into prognostic indicators of HCC revealed that portal vein tumor thrombosis precipitated portal vein hypertension, ruptures of esophageal and rectal varices, ascites, and ischemic liver damage [65]. In instances where tumor cells invaded the hepatic or portal veins, survival times were as brief as 2–3 months. This underscores the importance of identifying such invasions whenever feasible. The study, despite its short follow-up period, primarily reflects the dire prognosis and restricted survival times observed among patients. Rigorous analyses encompassing univariate and multivariate Cox models were conducted to factor in liver function, treatment, and comorbidities as prognostic determinants. The role of the hepatic artery as a prognostic factor has also emerged in gallbladder carcinoma. A study involving 71 patients who underwent radical resection for gallbladder carcinoma underscored a significant association between hepatic artery invasion and poorer prognosis compared to patients without such invasion. Multivariate analysis revealed invasion of the hepatic artery as the sole independent prognostic factor [69].

To specify, neoplasms that demonstrate LVI may have a better prognosis if this invasion is detected early. In the process of determining the treatment plan and/or the prognosis, different factors should be considered. In the case of endometrial and gallbladder carcinomas, detection of vascular invasion is associated with higher TNM staging [68,69]. Depending on the stage of the cancer, specific targeted therapies may be considered to prevent vascular invasion.

Within the spectrum of the studies reviewed in this article, some limitations are present, including sample size and staining techniques. The choice of staining technique and methodologies exerts an influence on the detection of venous invasion. A prognostic exploration of LVI in patients afflicted with esophageal SCC yielded findings that underscored the potential for staining techniques to yield varied detection outcomes for vascular invasion when juxtaposed with studies employing different techniques for the same cancer type. This study underscored the importance of segregating lymphatic vessel invasion from vascular invasion in prognostic assessments given their distinct prognostic significance [63]. While these studies may harbor analogous limitations, they collectively underscore the pivotal role of vascular and venous invasions in shaping tumor prognosis.

## 5. Conclusions

The literature shows an overwhelming agreement on the superiority of elastin stains alongside H&E over H&E alone for the detection rates of vascular (particularly venous) invasion in CRCs and other GI and non-GI malignancies. The use of elastin stains is significantly correlated with an increased risk of hematogenous spread, decreased relapse survival, and disease-free survival. The link between vascular invasion, mitotic index, and tumor size and stage also shows significant prognostic value. Findings also show that venous invasion can be crucial in assessing the risk of cancer-related comorbidities such as portal vein hypertension in HCC and gallbladder carcinoma. Although most studies agree on the enhanced prognostic value of elastin stains, this finding is not unanimous across the literature. Researchers report varying degrees of the prognostic significance that elastin stains have on the detection of venous invasion, with some studies reporting no predictive value. These findings reflect the complex association between venous invasion and its clinical value. Overall, there is a significant consensus on the superiority of elastin staining for detecting venous invasion, demonstrating a clear clinical value. Reports also show the enhanced efficiency of using elastin stains by showing an increased detection rate in venous invasion independent of the pathologist’s experience or level of sub-specialization. A connection between elastin stain detection rates and a decrease in the used resources and time required for detection has also been reported, further adding to the value of this technique.

Further research should focus on pinpointing the true prognostic value of vascular invasion detection with elastin stains across different malignancies. Additional research is also needed to determine the prognostic association between venous invasion and cancer-associated comorbidities. Ultimately, as knowledge of the prognostic value of elastin satins used for vascular (venous) invasion detection increases, this method can potentially improve therapeutic strategies and patient outcomes.

## Data Availability

No new data was created or analyzed in this study. Data sharing is not applicable to this article.
